# New York Letter

**Published:** 1898-04

**Authors:** Ogden C. Ludlow

**Affiliations:** 2309 Seventh Avenue; New York


### CORRESPONDENCE.

### NEW YORK LETTER.

New York, March 15, 1898.

*Editors Atlanta Medical and Surgical Journal:*

On the closing day of 1897, the Board of Health made the announcement—cheering or depressing, depending upon the point of view—that the city of New York had never been so healthy as during the year just past. The records show that the death-rate was only 19.62. Although we may infer from this that the "business" of the medical practitioner here has not been as good as he might desire, there is nothing to indicate that he has lost any of his old-time interest in medicine as a *study*. True, the once familiar figure of the "family doctor" has become a rare bird in these parts, but the activity—I had almost said the "pernicious activity"—of the specialists serves to keep alive a proper interest in our medical gatherings.

The following method of performing taxis in cases of strangulated hernia is offered by Dr. William B. De Garmo, who claims for it a very large proportion of successful reductions. His very large experience with this class of cases should give such a recommendation great weight. The instructions are, to take hold of the tumor and *draw it down*, in this way lengthening the part in the canal at the point of constriction. Then, without attempting to push it up, steady pressure is to be made on the tumor with the idea of forcing out its contents. If the patient is a child, it is also well to suspend the little one by one leg while taxis is being made.

Another surgical wrinkle that should be included in the next list of "Surgical Dont's" is in regard to the danger of using plain water, without salt, for intravenous injections. It is not difficult to suppose that in an emergency, a stock of normal saline solution not having been previously made ready, a surgeon might think that time was too precious to waste any of it in making up salt solution, and would accordingly use plain hot water. Dr. R. H. M. Dawbarn assures us that such a procedure is not only unwise, but exceedingly dangerous. As far back as 1890, in some experiments on animals, he accidentally discovered that an intravenous injection of non-saline solution would prove very rapidly fatal, and then he was told by the physiologists that this result was due to the rapid solvent action of non-saline water on the blood corpuscles.

Speaking of saline injections, reminds me of a method of treatment for various diseased conditions, which has become so popular here of late that it would deserve to be dubbed a "fad," were it not that such an appellation would give a wrong impression as to its value. I refer to continuous irrigation of the bowel. It has been the subject of papers, discussions and various demonstrations this winter. This is largely owing to the enthusiastic advocacy of the method by Dr. Robert Coleman Kemp, the inventor of several varieties of double-current rectal tubes. Dr. Kemp has not been satisfied with the rapidly accumulating clinical data in favor of the method, but has subjected it to critical tests in the physiological laboratory. Both the observations in the laboratory and those at the bedside seem to show conclusively that it is a convenient and potent method of *preventing* and combating shock, and of treating cases of suppression of urine, to say nothing of its beneficial action in certain diseased conditions found in both the male and female pelvis. A number of our surgeons make use of this form of rectal irrigation, as a routine measure, *during* all operations in which there is reason to believe shock will be produced, and some of them go so far as to say that it is equal, if not superior, to saline infusion in cases of profuse hemorrhage. There is, moreover, great unanimity of opinion regarding its power to arouse the kidneys to functional activity. Dr. E. H. Grandin says that he knows of no other method of treatment for cases of urinary toxemia, with impending eclampsia, that is equal to irrigation of the bowel with water at a temperature of 115° to 120° F. In one case of anuria that had lasted thirty-six hours, he succeeded in saving the woman's life by using in this way fifteen gallons of hot water.

While speaking of matters of surgical interest, it is but right to refer to the work of Dr. Willy Meyer with Schleich's new method of inducing general anesthesia. The fundamental principle of the method is, that the absorption of the anesthetic agent is regulated chiefly by the relation existing between the body temperature of the patient and the boiling point of the anesthetic. If, as in the case of chloroform, the boiling point is much higher than the body-temperature, the anesthetic will be absorbed in unnecessarily large quantity, and will overtax all the parenchymatous organs. Schleich's solutions are composed of varying proportions of chloroform, petroleum ether and sulphuric ether, thus giving control of the boiling point of the anesthetic agent. Dr. Meyer's experience with this new method comprises about one hundred cases, so that he is entitled to speak with more assurance than a number of others here who have tried it in a few instances only, but it must always be remembered that, in studying the effects of a general anesthetic, very positive conclusions should only be drawn from an experience, not of two or three hundred, but of several thousand cases. Dr. Meyer has found that the pulse remains of good quality, that there is no hypersecretion of mucus, that vomiting occurs in about 44 per cent. of the cases, and albuminura in 4 per cent., and that the return to consciousness has been more rapid than after either chloroform or ether. There has also been a notable freedom from consecutive bronchitis and pneumonia. Experience has shown that the best mode of administering the Schleich anesthetic solutions is by the use of an Esmarch mask covered with flannel and oil-silk, with a funnel so adapted to it that the solution may be made to drop on the mask at a given rate. The usual rate is one drop every four or five seconds, but it should be rather more rapid at the commencement. The chief difficulty experienced has been in securing in the market a specimen of petroleum ether having the requisite boiling point.

We have all heard a great deal about the injurious effects sometimes observed after the use of the X-rays, but I believe that Dr. James P. Tuttle, of New York, has the distinction of being the first who has been called upon to amputate a limb because of such an injury. It happened in this way. The patient was an old soldier who had long suffered from disease of the knee-joint. About three years ago, an operation, largely experimental, was done on him. It showed the presence of some floating bodies in the joint, but failed to reveal the exact nature of the painful affection from which he suffered. It did, however, give him complete relief for nearly three years. On the recurrence of the pain, his attending physician submitted the part to a long and searching examination with the X-rays. This caused no discomfort at the time, but at the end of about three weeks the part became red and painful, and in the course of two or three days very extensive sloughs had formed. Skin-grafting was resorted to, at first with a very gratifying result, but after five weeks the whole grafted area broke down. From this time on, the man's health failed rapidly, and he became addicted to the use of morphine. A consultation was held, and, as a result, the thigh was amputated. At the last report the patient was doing well.

This case is full of interest, not alone as showing the possible gravity of such accidents, but because of the important points brought out regarding the nature of these so-called burns. Dr. E. B. Bronson says that it is far from accurate to speak of these injuries as "burns," because unlike true burns, there are no immediate effects observable, there is a distinct and rather long interval between the exposure to the X-rays and the development of the local lesions, and the pathological process spreads from within outward. It should be noted also that these "burns" are not amenable to the same treatment as ordinary burns. Thus, Dr. Tuttle found that the external applications usually relied on to mitigate the patient's suffering, seem to actually increase the pain experienced in connection with X-ray burns.

Those who use this important adjunct of modern surgery will do well to remember that it is *claimed* that no such accidents have followed exposure to X-rays generated by a static machine, and that they have all been produced in connection with the Rhumkorff coil apparatus. It is also well to bear in mind that experience has shown that but few of these "burns" heal in less than nine months, and that the healing process often covers a period two or three times as long. The quickest cures in these cases, I believe, have been effected by Dr. Seneca D. Powell, who has observed healing occur in six or eight weeks in cases in which he had promptly excised the affected area. I infer that in neglected cases it is necessary to excise very deeply, although I have no specific information on this point. It is noteworthy that in the case of amputation of the thigh, examination of the knee-joint after the operation showed that the pathological process set up by the X-rays had extended down even to the capsular ligament of the joint.

As a result of a clinical study of the effects of diphtheria on the heart action, Dr. Henry Dwight Chapin has called special attention to a sudden and peculiar slowing of the pulse which is of considerable prognostic importance. He finds that this retardation is observed chiefly in the grave septic cases, irrespective of whether the pulse has previously been slow or rapid. A slow pulse, without other symptoms, is not necessarily of grave omen, but when the slowing is sudden and extreme, death invariably occurs. In one case, which he reports, that of a boy of five years, the pulse suddenly dropped to 28 per minute, and in spite of free stimulation the child died in two days. Dr. J. E. Winters contends that a slow pulse in diphtheria has been considered by all authorities on that disease to be of very rare occurrence, and from its more frequent occurrence at the present time, and especially among cases receiving the antitoxin treatment, he is disposed to infer that there is some direct connection between the two. Dr. H. W. Berg, on the other hand, asserts that he met with this peculiar symptom many years before diphtheria antitoxin was known or thought of.

A question which almost daily confronts the medical practitioner is, What importance is to be attached to the finding of albumin and casts in the urine? The answers vary greatly, but it is probable that in the great majority of instances, the tendency is to take too gloomy and absolute a view. Dr. William Henry Porter claims that hyaline casts are not uncommonly found in the urine of gross feeders, and in those who exhibit a tendency to the over-production of uric acid; and he explains their presence in the urine by saying that they mean little more than that an isomeric albumin has been excreted through the renal cells and has been precipitated by the uric acid. But if epithelial or nucleated casts are present there is good reason for believing that the kidneys are really diseased. Regarding albuminuria, the same observer maintains that its significance depends very largely on the habits of the individual. If the patient is an animal-feeder, he looks upon the prognosis as much more favorable than where the individual is largely vegetarian, or partakes freely of fruits. ODGEN C. LUDLOW, M.D.

*2309 Seventh Avenue.*

